# Controlled Delivery of Sonic Hedgehog Morphogen and Its Potential for Cardiac Repair

**DOI:** 10.1371/journal.pone.0063075

**Published:** 2013-05-14

**Authors:** Noah Ray Johnson, Yadong Wang

**Affiliations:** 1 Department of Bioengineering, University of Pittsburgh, Pittsburgh, Pennsylvania, United States of America; 2 McGowan Institute for Regenerative Medicine, Pittsburgh, Pennsylvania, United States of America; 3 Department of Chemical and Petroleum Engineering, University of Pittsburgh, Pittsburgh, Pennsylvania, United States of America; 4 Department of Surgery, University of Pittsburgh, Pittsburgh, Pennsylvania, United States of America; Northwestern University, United States of America

## Abstract

The morphogen Sonic hedgehog (Shh) holds great promise for repair or regeneration of tissues suffering ischemic injury, however clinical translation is limited by its short half-life in the body. Here, we describe a coacervate delivery system which incorporates Shh, protects it from degradation, and sustains its release for at least 3 weeks. Shh released from the coacervate stimulates cardiac fibroblasts to upregulate the expression of multiple trophic factors including VEGF, SDF-1α, IGF-1, and Shh itself, for at least 48 hours. Shh coacervate also demonstrates cytoprotective effects for cardiomyocytes in a hydrogen peroxide-induced oxidative stress environment. In each of these studies the bioactivity of the Shh coacervate is enhanced compared to free Shh. These results warrant further investigation of the *in vivo* efficacy of Shh coacervate for cardiac repair.

## Introduction

Heart failure afflicts 5.7 million Americans, a prevalence that is expected to rise 25% by 2030 [1]. There is an urgent need for preventative and regenerative therapies for this deadly disease, for which current treatments only seek to slow its progression [2]. The best therapy may involve “master switch” agents which activate multiple downstream pathways [3]. Sonic hedgehog (Shh) is one such master regulator, acting as a powerful morphogen during development when it typically influences cell fate but can also affect cell growth and survival [4], [5]. Shh signaling remains active in the adult mammalian heart at very low levels though it is upregulated during ischemic heart injury such as myocardial infarction (MI) and congestive heart failure [6], [7]. Currently, damage to the adult heart is considered permanent as cardiomyocytes have limited proliferative capability and fibrosis occurs post-MI instead of healing [2], [8]. However, during development, when cardiac tissue patterning is controlled by powerful morphogens such as Shh [9], the embryonic mammalian heart briefly displays the ability to regenerate itself [10]. Temporarily recapitulating this embryonic signaling environment through exogenous application of cardiac morphogen Shh is therefore a possible approach towards repair of the damaged adult heart.

Shh has previously been applied to the heart as a free protein. Direct injection has been shown to restore blood flow in a critical hindlimb ischemia model [11]. However, this approach is highly inefficient due to short morphogen half-lives in the body [12] and therefore requires multiple injections. High dosage is also necessary to elicit a desired effect which is expensive and may carry similar safety concerns to gene therapy. Investigations using gene therapy [7], [13] and transduced cell therapy [14] have been shown to improve cardiac function following MI. However, both of these approaches carry high risk of inducing tumor formation, especially considering the potency of morphogens [15], and thus trials have not progressed beyond large animal models. On the contrary, a controlled delivery approach provides stabilization and protection of Shh and can release it slowly to maintain a constant local concentration within the therapeutic range.

Here we introduce a controlled delivery system comprised of heparin and a synthetic polycation that interact polyvalently and phase separate from water to form sub-micron sized spherical droplets. These emulsion-like aggregations are referred to as a “coacervate”. Shh is incorporated into the coacervate by high-affinity binding to heparin and is then released in a slow and sustained fashion. We demonstrate that Shh delivered by the coacervate can have multiple beneficial effects on cardiac cells, stimulating trophic factor expression and affording cytoprotection from oxidative stress, and may therefore have the potential to protect the heart from ischemia or promote tissue regeneration following insult. This is, to the best of our knowledge, the first investigation of controlled delivery of Shh for cardiac repair.

## Materials and Methods

### Ethics statement

Animals were cared for in compliance with a protocol approved by the Institutional Animal Care and Use Committee of the University of Pittsburgh, following NIH guidelines for the care and use of laboratory animals (NIH publication No. 85–23 rev. 1985).

### Shh coacervate preparation

Poly(ethylene argininylaspartate diglyceride) (PEAD) was synthesized as previously described [16]. PEAD and clinical-grade heparin (Scientific Protein Labs, Waunakee, WI) were each dissolved in 0.9% saline at 10 mg ml^−1^ and 0.22 µm filter-sterilized. Heparin was initially complexed with recombinant mouse Shh (N-terminus peptide; R&D Systems, Minneapolis, MN), then PEAD was added. Self-assembly of PEAD and heparin:Shh immediately precipitated the ternary complex out of solution to form the Shh coacervate.

### Shh coacervate imaging

Shh was fluorescently labeled using Alexa Fluor 488 Protein Labeling Kit (Invitrogen, Carlsbad, CA). A fluorescently labeled Shh coacervate was prepared in water with 0.2 ng fluorescently labeled Shh, 100 µg heparin, and 500 µg PEAD. The coacervate was added to a 96-well plate and imaged using an Eclipse Ti inverted microscope (Nikon, Tokyo, Japan).

### Shh release profile

200 µl Shh coacervate containing 10 µg heparin, 100 ng Shh, and 50 µg PEAD was prepared in 0.9% sterile saline and was then pelleted by centrifugation at 12,100 g for 5 min. The supernatant was aspirated and stored at −80°C and 200 µl fresh saline was replaced to cover the pellet. The sampling procedure was repeated on day 1, 4, 7, 10, 14, and 21 for three separate tubes. Released Shh in the fractions was quantified by DuoSet ELISA kit (R&D Systems) according to the manufacturer's instructions.

### Cardiac cell isolation

Primary cardiac cells were isolated from 1–2 day old Sprague-Dawley rat pups (Jackson Labs, Bar Harbor, ME). The pups' hearts were excised aseptically and the Worthington Neonatal Cardiomyocyte Isolation System (Worthington, Lakewood, NJ) was used following the manufacturer's instructions. Two pre-plates of 1.5 h each were used to separate cardiac fibroblasts (fast-adhering cells) from cardiomyocytes (slow-adhering cells). The purified cardiomyocyte population was seeded into 24-well plates coated with 1 µg cm^−2^ fibronectin (Millipore, Billerica, MA) at 1.5×10^5^ myocytes per well for experiments. Cardiomyocytes were cultured in DMEM/F12 50/50 supplemented with 5% fetal bovine serum (FBS), ITS supplement (Sigma, St. Louis, MO), and 100 µM 5-bromo-2′-deoxyuridine (BrdU; Sigma) to inhibit proliferation of any remaining fibroblasts. Cardiac fibroblasts from the first pre-plate were cultured in DMEM supplemented with 10% FBS through 3 passages before use in experiments.

### Immunofluorescent staining

Immediately after isolation, purity of cardiac cell populations was analyzed by immunostaining using a mouse anti-myosin heavy chain (MHC) antibody (1∶80, Millipore) followed by goat anti-mouse IgG Alexa Fluor 594-conjugated secondary antibody (1∶200, Invitrogen) and counterstained with DAPI. All images were taken using an Eclipse Ti inverted microscope (Nikon).

### Cardiac fibroblast growth factor signaling

Isolated neonatal rat cardiac fibroblasts were used at passage 4. 5×10^4^ cells were seeded per well in a 24-well plate and cultured to 90% confluency. Cells were then washed once with DPBS and 500 µl “stimulation media” containing 1 mg ml^−1^ Shh free or in the coacervate, or normal culture media was added. After 6, 12, 24, 48 h the conditioned media from two wells per group was removed and frozen. Indirect ELISA was run using rabbit polyclonal antibodies against VEGF (1∶50, Peprotech, Rocky Hill, NJ), SDF-1α (1∶30, Santa Cruz Biotech, Santa Cruz, CA), and IGF-1 (1∶30, Santa Cruz Biotech). Results were normalized to total protein content, determined using Pierce 660 nm Protein Assay (Thermo Fisher Scientific, Waltham, MA). For Shh quantification, western blot was employed with samples being denatured and reduced. Lacking a well-accepted protein loading control for conditioned media, total protein assay results were used to determine appropriate loading volumes for each sample, such that 47.5 µg protein was loaded to each well. Following transfer, the PVDF membrane was incubated for 5 m in Ponceau-S staining solution (Sigma), rinsed in DI water and imaged. Immunoblotting was then performed using a rabbit anti-human Shh polyclonal antibody (1∶200, Santa Cruz Biotech) followed by a peroxidase conjugated anti-rabbit IgG antibody (Sigma). Band intensities were measured using NIH Image J Version 1.46r software.

### Cardiomyocyte oxidative stress-induced apoptosis

Isolated cardiomyocytes were cultured for 2 d after which cells were spontaneously contracting at approximately 1 beat per second. Cells were pre-treated for 48 h with 500 ng ml^−1^ Shh or recombinant human fibroblast growth factor-2 (FGF2; Peprotech) as either free protein or delivered by the coacervate. The FGF2 coacervate was prepared similarly to the Shh coacervate. Cells were then exposed to 200 µM H_2_O_2_ in serum-free media to induce oxidative stress. Two control groups received no growth factor pre-treatment and then one received H_2_O_2_ and one did not. After 2 h, cells were harvested, lysed, and assayed using EnzChek Caspase-3 Kit #2 (Invitrogen). Immediately before harvesting the cardiomyocytes, videos were taken in the center of the wells using an Eclipse Ti inverted microscope (Nikon) using standard light microscopy settings.

### Statistical analysis

Statistical analysis was performed using SPSS 16.0 software (SPSS Inc, Chicago, IL). Data was tested for normality and equal variance before analysis. Statistical differences between groups were calculated using one-way analysis of variance (ANOVA) followed by Tukey post-hoc testing. Differences were considered significant at *P*<0.05.

## Results

### Shh coacervate characterization and release

**Figure 1:**
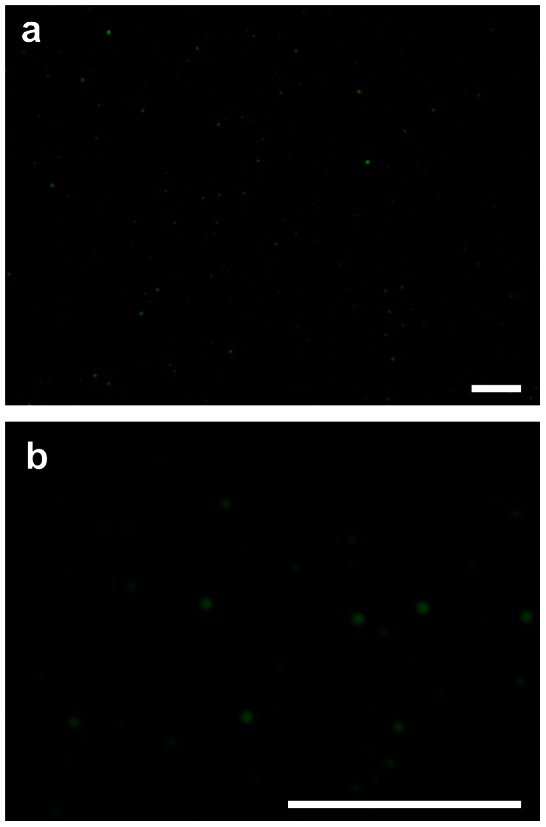
Shh coacervate imaging. Alexa Fluor 488-labeled Shh was incorporated into the coacervate in water and imaged by fluorescence microscopy. (**a**) 10× magnification. (**b**) 40× magnification. Scale bars = 100 µm.

**Figure 2:**
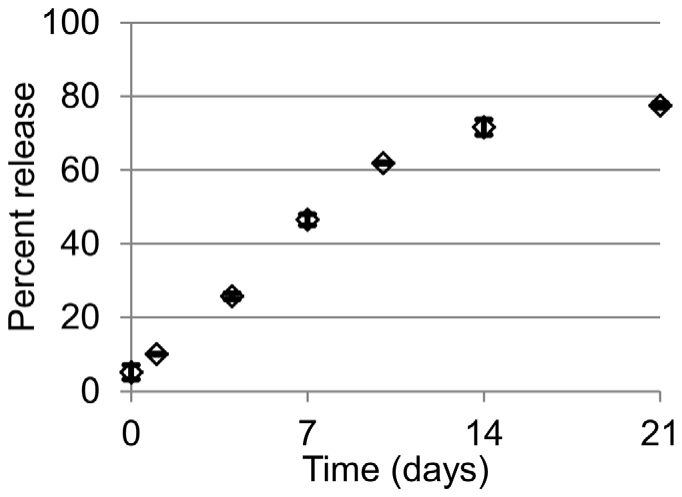
Shh release profile. *In vitro* release of Shh from the coacervate into saline over 21 days, quantified by sandwich ELISA. Percent release is relative to total amount loaded. Bars indicate means ± SD.

Fluorescent imaging of the Shh coacervate revealed round droplets ranging from 0.5–10 µm in size (Fig. 1). Shh release from the coacervate into saline was sustained for at least 3 weeks with 80% being released in total (Fig. 2). Less than 5% Shh was detected in the supernatant immediately after forming the coacervate and pelleting, therefore loading efficiency is greater than 95%. There was effectively no initial burst release. Of the 100 ng loaded, approximately 6 ng day^−1^ was released until day 10, then slowing to around 2 ng day^−1^ until day 21.

### Shh coacervate upregulates growth factor secretion by cardiac fibroblasts

**Figure 3:**
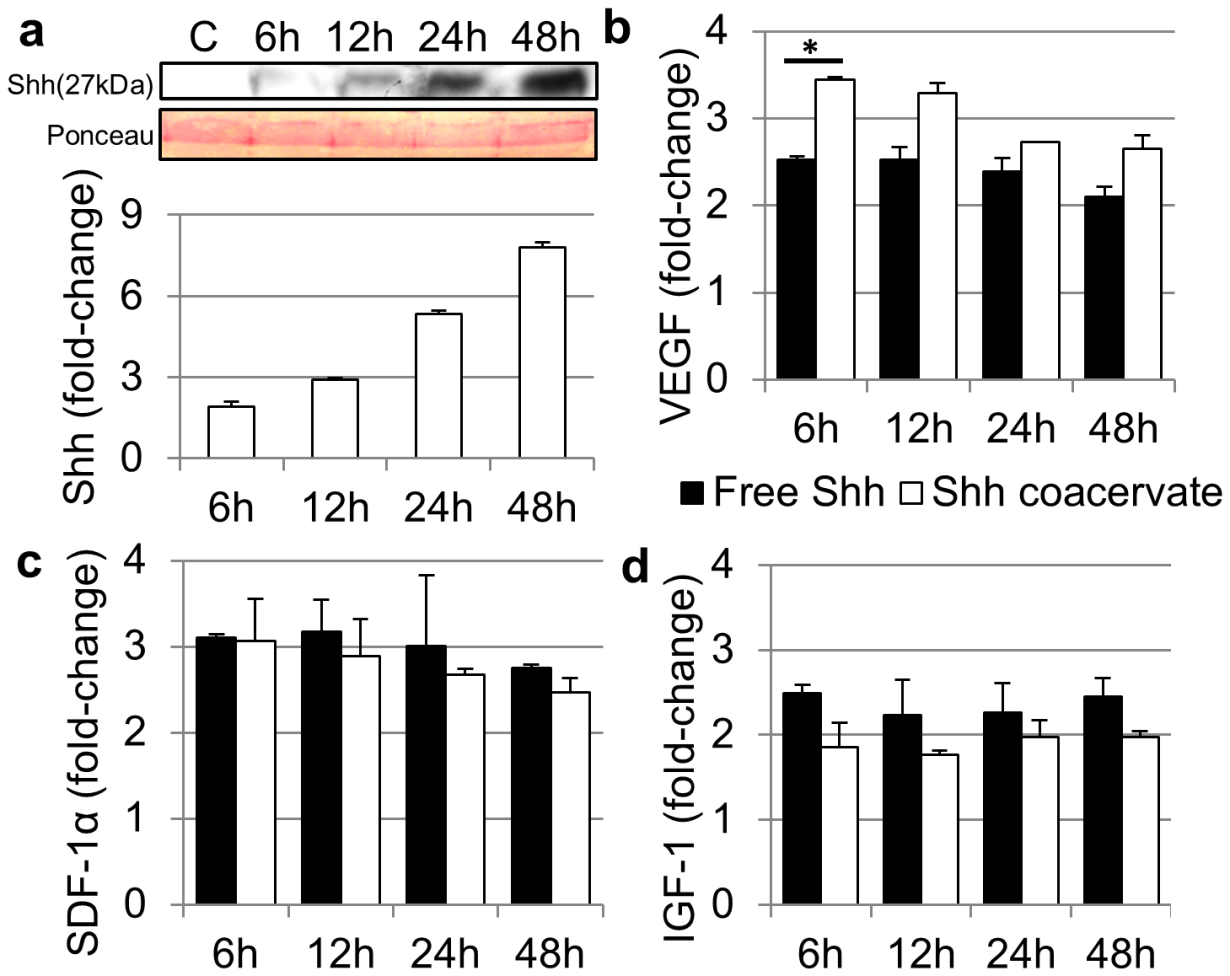
Shh-stimulated cardiac fibroblast signaling. Near-confluent cardiac fibroblasts were incubated with Shh, free or in the coacervate, and growth factor levels in the conditioned media were assessed after 6, 12, 24, and 48 h. Data is presented as a fold-change from the stimulation media (C). Bars indicate means ± SD. (**a**) Quantification of Shh concentration in the cardiac fibroblast-conditioned media by western blot. Ponceau-S staining of protein bands near 27 kDa is shown as the loading control. (**b**) Quantification of VEGF in the cardiac fibroblast-conditioned media by indirect ELISA, *P<0.05. (**c**) Quantification of SDF-1α in the cardiac fibroblast-conditioned media by indirect ELISA. (**d**) Quantification of IGF-1 in the cardiac fibroblast-conditioned media by indirect ELISA.

Isolated neonatal rat cardiac fibroblasts were stimulated with Shh, free or in the coacervate, and analyzed for expression of various growth factors over 2 days. Shh itself was detected in increasing concentrations, up to 7.8 times the initially added amount by 48 h (Fig. 3a). VEGF, SDF-1α, and IGF-1 were at 2- to 4-fold higher levels by 6 hours after stimulation with both free Shh and Shh coacervate and remained upregulated for 48 h (Fig. 3b–d). The VEGF level at the 6 hour timepoint stimulated by Shh coacervate was significantly greater than that stimulated by free Shh. Conditioned media from cells treated with normal culture media had undetectable concentrations of all growth factors tested at every timepoint (data not shown).

### Shh coacervate protects cardiomyocytes from apoptosis

**Figure 4:**
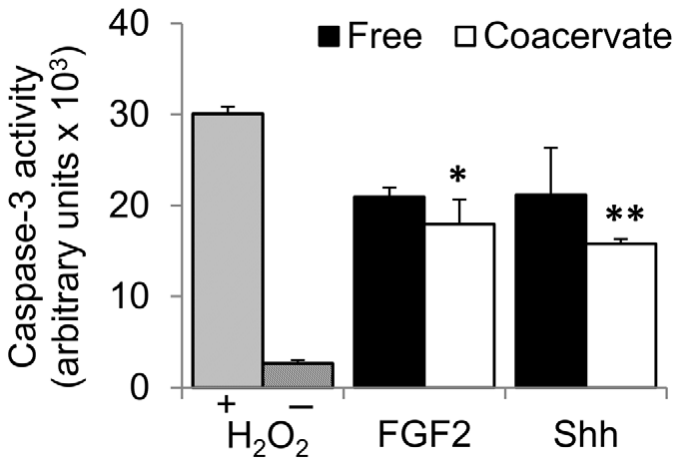
Cytoprotection of cardiomyocytes from oxidative stress-induced apoptosis. Myocytes were pre-treated for 48 h with FGF2 or Shh, free or delivered in the coacervate. Cells were then exposed to oxidative stress conditions with H_2_O_2_ for 2 h. Two control groups were not pre-treated with any growth factors and then one received H_2_O_2_ for 2 h (+) and one did not (−). Cell apoptosis levels were measured by caspase-3 activity. *P<0.05, **P<0.01 compared to the +H_2_O_2_ control group.

Caspase-3 is a well-known indicator of cell apoptosis due to oxidative stress, mimicked here with 200 µM H_2_O_2_ in culture media. This concentration of H_2_O_2_ is higher than has been previously reported to induce myocyte apoptosis [17]. Cardiac cell isolation yielded a highly pure population of primary neonatal rat cardiomyocytes (Fig. S1). Myocytes pre-treated with FGF2 coacervate or Shh coacervate showed significantly lower levels of apoptosis compared to the H_2_O_2_ treated control, but higher levels than the control group not treated with H_2_O_2_ (Fig. 4). Additionally, the difference in means compared to the H_2_O_2_ treated control was more statistically significant for Shh coacervate than for FGF2 coacervate (p = 0.003 vs. p = 0.012). Incubation with free FGF2 or Shh did reduce caspase-3 levels, however these differences were not statistically significant (p = 0.094 and p = 0.083) from the H_2_O_2_ treated control. Videos taken at the experiment endpoint show that cardiomyocytes were beating normally in the non-H_2_O_2_ treated control (Video S1), but rapidly and irregularly in the Free Shh group (Video S2) and Shh coacervate group (Video S3). Additionally, more cells were beating in the Shh coacervate group than in the Free Shh group in accordance with our caspase-3 results.

## Discussion

Ischemic damage to the heart may be mitigated by Shh through three key mechanisms: i) Angiogenesis- the sprouting of new blood vessels from pre-existing ones, ii) Cytoprotection of cardiomyocytes under oxidative stress, and iii) Recruitment of cardiac progenitor cells.

Shh-induced neovascularization is a result of upregulated paracrine signaling by fibroblasts of several angiogenic factors including vascular endothelial growth factor (VEGF) and angiopoetin-1 [11], [18], [19]. It has also been shown to increase the contribution of bone marrow-derived endothelial progenitor cells (EPCs) to the neovascularization process [19]–[21]. Additionally, recent reports have revealed that Shh acts through the Rho-associated protein kinase (ROCK) pathway, rather than its traditional Gli-dependent pathway, to stimulate expression of matrix metalloproteinase-9 (MMP-9) and osteopontin (OPN) in endothelial cells [22], [23]. These multiple complex roles of Shh underscore its importance in ischemic revascularization. In the present work we show that the Shh coacervate can stimulate cardiac fibroblasts to significantly upregulate expression of VEGF. Furthermore, Shh coacervate acutely (6 hour timepoint) stimulated significantly more VEGF production than free Shh. This may be attributed to enhanced morphogen bioactivity when delivered by the coacervate, as discussed later. *In vivo*, this upregulated angiogenic growth factor expression may induce angiogenesis to re-vascularize the ischemic myocardium.

The cytoprotective effects Shh displays for cardiomyocytes have also been attributed to multiple mechanisms. One contributor is insulin-like growth factor (IGF-1) which has been shown to be upregulated in Shh-stimulated cardiac fibroblasts [7] and bone marrow-derived cells [24]. IGF-1 interferes with the transcription of angiotensin II, thereby preventing the formation of reactive oxygen species during oxidative stress [25], [26]. A more direct anti-apoptotic role of Shh has also been demonstrated *in vitro* for cardiomyocytes, which express the Shh receptor Patched-1 [7]. Our results seem to confirm this direct role, as recombinant Shh protein applied alone to stressed cardiomyocytes reduced cell apoptosis. Yet we must still consider that the cardiomyocyte population was not entirely pure and may have contained a small number of fibroblasts. Upregulated secretion of IGF-1 by these fibroblasts may have therefore played a role as well. FGF2 was included as a positive control in apoptosis experiments as it is a well-known survival factor for numerous cell types [27]–[29] including cardiomyocytes [30], and we have experience delivering it with the coacervate [31]–[33]. We observed the coacervate to enhance the bioactivity of both FGF2 and Shh compared to either factor in free form. We also found that Shh coacervate protected stressed cardiomyocytes better than FGF2 coacervate. This is significant because FGF2 expression by fibroblasts has actually been shown to be downregulated in response to Shh stimulation [11]. The anti-apoptotic effects of Shh must therefore be ascribed to non-FGF2-related pathways.

Stromal cell-derived factor-1 (SDF-1), also known as CXCL12, is a trafficking chemokine for stem and progenitor cells [34]. It has been shown to act in local recruitment of cardiac progenitor cells [35]–[37], as well as EPCs [38] and smooth muscle progenitor cells [39]. It is well accepted that SDF-1 plays a pivotal role in stem and progenitor cell homing to sites of injury [40]. We prove here that Shh coacervate can upregulate production of SDF-1α at least as well as equal dose of free Shh, therefore the bioactivity of released Shh is well maintained. *In vivo*, high local levels of SDF-1α may encourage recruitment of blood derived EPCs to aid in neovascularization, and of cardiac progenitor cells to engender myocardial tissue repair.

Clearly, Shh acts as a “master switch” agent in many tissues including the heart, activating multiple downstream pathways in response to injury. Shh has been shown to activate the ERK1/2 [41], PKC [14], ROCK [22], and PI3K/Akt [20] signaling pathways, among others. Shh morphogen may therefore provide benefits similar to co-delivery of multiple different growth factors. However, a multi-factor approach would require extensive optimization of growth factor dosage and release rate, while Shh stimulates a natural healing environment, similar to the embryonic state when cardiac regeneration is possible. Finally, we also observed upregulated Shh expression by cardiac fibroblasts in response to stimulation with Shh, a positive autoregulation that has been previously reported [7].

Regarding the role the coacervate plays in enhancing the effects of Shh, we point to heparin as a major player. Proteoglycans associated with the extracellular matrix or cell surface play a vital role in the transduction of cell signaling pathways [42]. Heparin binds many growth factors, cytokines, and morphgens, stabilizes and protects them, and in some cases modulates their activity [43], [44]. It has been shown to interact with a growth factor molecule and its corresponding cell receptor simultaneously, forming a stable ternary complex which facilitates signal activation [45]. Heparan sulfate proteoglycans have also been shown to play a vital role in developmental patterning and specifically in regulating Hedgehog distribution and pathway activation [46], [47]. Heparin binds Shh with high affinity (K_d_ = 99 nM) [48] and may inherently enhance its bioactivity by mimicking the *in situ* signaling environment. Indeed, the biological activity of Shh has been shown to be directly related to its affinity for heparin [48]. We suggest that utilizing heparin in our delivery system may thereby potentiate the activity of Shh. This is supported by our previous reports using the coacervate to deliver FGF2 [32], [33], neuronal growth factor [31], and heparin-binding EGF-like growth factor [49]. However, the efficacy of the coacervate at maintaining or enhancing the bioactivity of Shh in an *in vivo* environment requires further evaluation.

The activity of bound factors is further preserved within the coacervate, which is in a separate phase from water. This isolation provides further protection from proteases. The heparin:Shh complex is soluble in water and if injected *in vivo* would therefore be quickly diluted and removed from the injection site. To maintain the complex locally, an insoluble coacervate is formed using a synthetic polycation, PEAD. This ariginine-based polycation was designed to imitate the highly cationic heparin-binding domain [45]. Heparin-bound factors are incorporated into the coacervate and protected from proteolysis and denaturation. Release from the coacervate is based on slow hydrolysis of the polycation [16], as well as dissolution of the complex in an ionic environment. We expect the release of Shh from the coacervate to be accelerated *in vivo* in in the presence of enzymes. We have previously shown this polycation to be highly biocompatible and to complex with heparin to control the release of many different growth factors [16]. The results presented here suggest that a simple liquid coacervate has high potential as a system of controlled delivery for heparin-binding morphogens, in addition to growth factors.

## Conclusions

The results of this study indicate that PEAD-heparin coacervate can load Shh with high efficiency and sustain its release for at least 21 days. Shh released from the coacervate displays bioactivity equal to or higher than that of free Shh. We demonstrate the ability of Shh coacervate to protect cardiomyocytes from oxidative stress and to upregulate secretion of multiple growth factors by cardiac fibroblasts. These results warrant further investigation of Shh coacervate for treating cardiac ischemia.

## Supporting Information

Figure S1 **Neonatal rat cardiac cell populations were separated by pre-plate technique.** Slow-adhering cardiomyocytes did not adhere to the pre-plate while fast-adhering cardiac fibroblasts did adhere. Both populations were immunofluorescent stained for myosin heavy chain (MHC) muscle cell marker (red) and counterstained with DAPI for cell nuclei (blue). Scale bars = 100 µm.
(TIF)

Video S1 **Cardiomyocytes in the – H_2_O_2_ control group not receiving any Shh pre-treatment and not treated with H_2_O_2_ in the apoptosis assay.**
(WMV)

Video S2 **Cardiomyocytes in the Free Shh group pre-treated for 48 h with 500 ng ml^−1^ free Shh protein and then treated with H_2_O_2_ for 2 h in the apoptosis assay.**
(WMV)

Video S3 **Cardiomyocytes in the Shh coacervate group pre-treated for 48 h with 500 ng ml^−1^ Shh in the coacervate and then treated with H_2_O_2_ for 2 h in the apoptosis assay.**
(WMV)
