# Supplementary figures and images for: Controlled Delivery of Sonic Hedgehog Morphogen and Its Potential for Cardiac Repair
Source: PLoS One. 2013 May 14;8(5):e63075. doi: 10.1371/journal.pone.0063075 (PMC3653884; doi:10.1371/journal.pone.0063075)

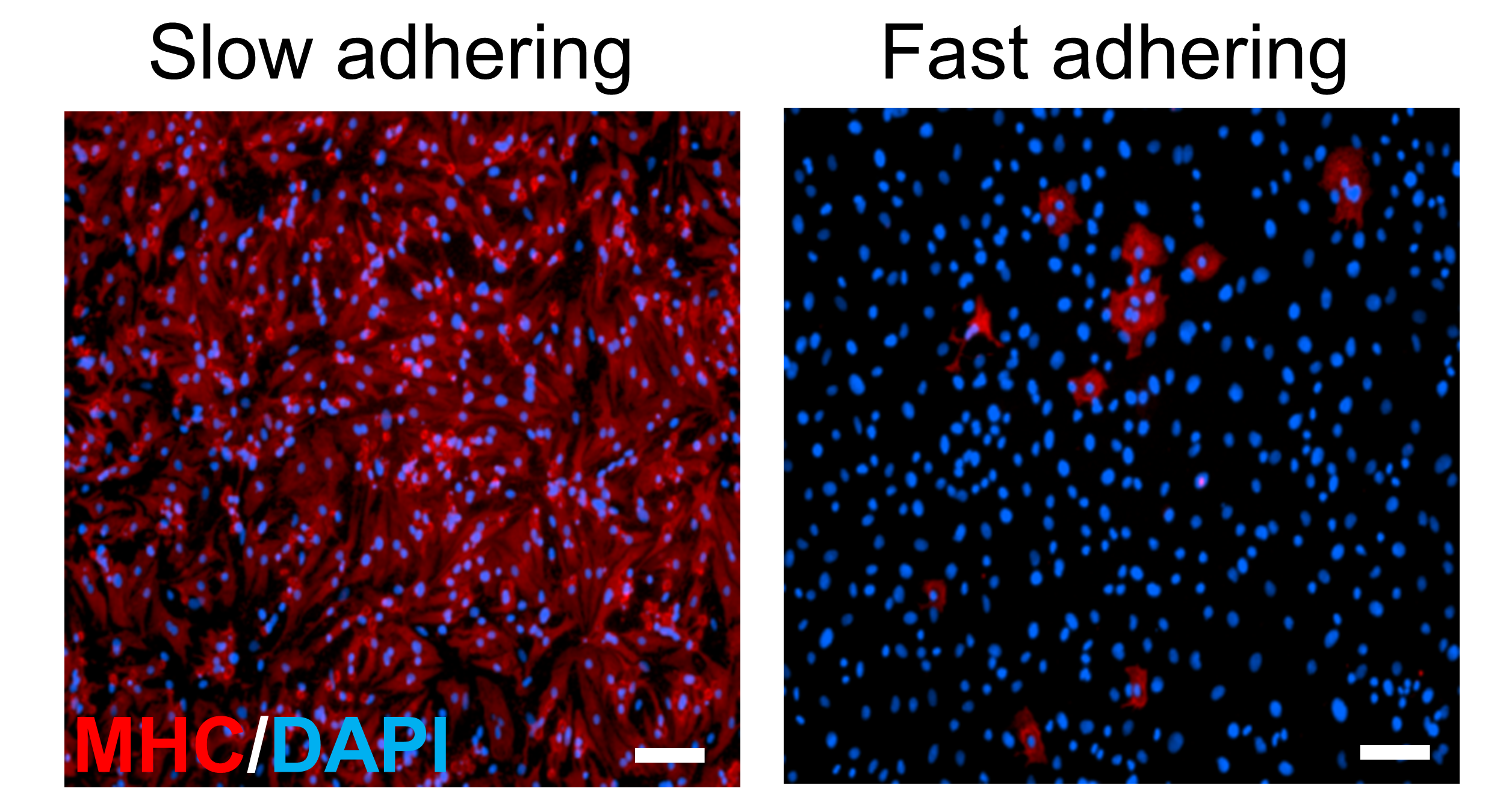

Supplement: Figure S1 — Neonatal rat cardiac cell populations were separated by pre-plate technique. Slow-adhering cardiomyocytes did not adhere to the pre-plate while fast-adhering cardiac fibroblasts did adhere. Both populations were immunofluorescent stained for myosin heavy chain (MHC) muscle cell marker (red) and counterstained with DAPI for cell nuclei (blue). Scale bars = 100 µm. (TIF) [file pone.0063075.s001.tif]
